# 4-Phenylbutyric acid protects islet β cell against cellular damage induced by glucocorticoids

**DOI:** 10.1007/s11033-021-06211-5

**Published:** 2021-02-10

**Authors:** Xueling Zhou, Yilin Xu, Yong Gu, Min Sun

**Affiliations:** 1 Department of Endocrinology, The First Affiliated Hospital of Nanjing Medical University, Nanjing, 210029 China; 2 Department of Endocrinology, Maanshan People’s Hospital, Maanshan, 243000 China; 3 Department of Nephrology, The Second Affiliated Hospital of Soochow University, 1055 Sanxiang Road, Suzhou, 215008 China

**Keywords:** Glucocorticoids, MIN6, Endoplasmic reticulum stress, 4-Phenylbutyric acid

## Abstract

This study, using the MIN6 cell line, examines the effect of glucocorticoids (GCs) on the expression and protein levels of endoplasmic reticulum stress (ERS) related genes. Furthermore, we evaluated the protective role of 4-phenylbutyric acid (4-PBA) on the aforesaid GCs induced changes. Pancreatic islet MIN6 cells were treated with dexamethasone (DEX) at distinct concentrations (0.1 μmol/L and 0.5 μmol/L) for different periods (1 h, 4 h, 12 h, and 24 h). The mRNA and protein levels of ERS related genes were measured using real-time qPCR (qRT-PCR) and western blotting. Similar evaluations were also carried out for the cells treated with 4-PBA combined with DEX. Upon DEX intervention which induces the unfolded protein response (UPR), the expression levels of BIP, ATF6, IRE1, and PERK increased in the MIN6 cells, both in concentration and time-dependent manner. Similarly, ERS associated gene *CHOP*, which is involved in the apoptotic pathway, also showed increased levels both in concentration and time-dependent manner. However, treatment with 4-PBA decreased the expression levels of ERS related proteins. Quantitative analysis found that all these results were statistically significant (*P* < 0.05). GCs markedly activates the ERS in the MIN6 cell line in vitro, however, this effect can be significantly alleviated upon treatment with 4-PBA.

**Supplementary Information:**

The online version contains supplementary material available at 10.1007/s11033-021-06211-5.

## Introduction

Diabetes mellitus, a pressing and growing health problem that affects over 463 million adults (20–79 years old) worldwide has become a global epidemic [1]. The pathogenesis of diabetes is a very complex phenomenon. In addition to genetic and environmental factors, abnormal secretion of insulin counter-regulatory hormones such as glucocorticoids (GCs) is also one of the mechanisms of diabetes pathogenesis.

GCs have a wide range of physiological effects, including regulation of glucose and lipid metabolism, lipolysis, liver gluconeogenesis, amino acid mobilization, and reduced skeletal muscle glucose uptake [2]. GCs function by multiple regulatory mechanisms for glucose metabolism. Mainly, they induce insulin resistance by inhibiting the insulin signal transduction pathway [3]. Besides, GCs may also affect insulin secretion from islet β-cells by regulating the concentration and distribution of cellular calcium ions [4, 5].

Pancreatic β-cells, the sites of insulin synthesis, have abundant endoplasmic reticulum (ER) structures. For maintaining cell viability and function, steady-state maintenance of ER is vital. But, a variety of reasons can lead to disorders of endoplasmic reticulum balance, known as "ER stress" (ERS) [6]. The ERS is an important link in the development of diabetes in the islet β cells [7]. Usually, when the accumulation of misfolded proteins in the ER reaches beyond a critical threshold, β-cells enable various adaptation mechanisms to match the function of the ER according to current cellular needs. This response is known as the UPR [8]. The UPR is mainly mediated by three signaling pathways, namely inositol-requiring enzyme 1 (IRE1); RNA-dependent protein kinase R-like ER kinase (PERK); and activating transcription factor 6 (ATF6) signal network [9]. Factors, such as increased unfolded/misfolded protein, Ca^2+^ depletion, or enhanced insulin biosynthesis affect ER homeostasis of islet β cells. This triggers the release of BIP, a major ER chaperone protein to activate UPR [10]. Simultaneously, long-term ERS may also induce apoptosis. The C/EBP homologous protein (CHOP), also known as DNA damage-inducible transcript 3 (DDIT3), plays an important pro-apoptotic role in the ERS-mediated apoptosis [11].

4-Phenylbutyric acid (4-PBA) is a non-selective chemical small molecule with a chaperone-like effect in increasing protein stability. It helps unfolded protein remodeling and has been approved by the U.S. Food and Drug Administration (FDA) for the prevention and treatment of diseases caused by the accumulation of misfolded proteins such as in motor neuron disease. Moreover, researches have also shown that 4-PBA can attenuate the GCs induced apoptosis of MC3T3-E1 osteoblast-like cells and inhibit ER stress and mitochondrial dysfunction [12].

In this study, using mouse insulinoma cell line MIN6, the effects of DEX which is a GC on ER-related proteins and respective insulin secretion-related genes (GLU2 and PDX1) were investigated in vitro. Also, we explored whether 4-PBA would affect the aforesaid regulation and if so, can it provide new targets for the prevention and treatment of diabetes, especially in those with abnormal GCs secretion.

## Materials and methods

### Cells and culture

The mouse insulinoma cell line MIN6 (a kind gift from Professor Zhuoxian Meng of Zhejiang University) was grown in 85% DMEM medium (Gibico) containing 25 mM glucose supplemented with 15% fetal calf serum (Gibico), 0.5% β-Mercaptoethanol (Gibico) and 0.2% antibiotics (Penicillin and Streptomycin, Gibico) at 37 °C in a humidified atmosphere containing 5% CO_2_. For preparation for DEX and 4-PBA, see supplementary material.

### Pharmacological intervention

The experimental groupings were as follows: the control group MIN6 was left untreated; the DEX group MIN6 cell line was treated with 0.1 µmol/L and 0.5 µmol/L DEX (Sigma, USA), and they all intervened for 1 h, 4 h, 12 h, and 24 h. In the 4-PBA + DEX group, DEX was co-incubated with 2.5 mmol/L 4-PBA (Sigma, USA). The 4-PBA group was only treated with 2.5 mmol/L 4-PBA.

### Real-time quantitative PCR (qRT-PCR)

The total cell RNA was extracted using the Trizol reagent kit. The concentration and purity of the total RNA were measured and it was reverse-transcribed into cDNA for analysis using the *qRT-PCR*. The comparative Ct (2 − ΔΔCt) method was used to calculate the relative mRNA amounts.

The primer sequences used in the experiments were as follows: β-actin upstream:5′-CGGGGACCTGACTGACTACC-3′ and downstream: 5′-AGGAAGGCTGGAAGAGTGC-3′; BIP upstream: 5′-AGGACAAGAAGGAGGATGTGGG-3′ and downstream: 5′-ACCGAAGGGTCATTCCAAGTG-3′; ATF6 upstream: 5′-TGGGCAGGACTATGAAGTAATG-3′ and downstream: 5′-CAACGACTCAGGGATGGTGCTG-3′; PERK upstream: 5′-CGATCAAATGGAAGCCCTTA-3′, and downstream: 5′-TGCGGATGTTCTTGCTGTAG-3′; IRE-1 upstream: 5′-AGTATTCCACCAGCCTCTATGC-3′, and downstream: 5′-CACACACTCTCCTTTGTCTCCA-3′; CHOP upstream: 5′- TTCACTACTCTTGACCCTGCGTC -3′ and downstream: 5′- CACTGACCACTCTGTTTC-3′; PDX1 upstream: 5′-CGGACATCTCCCCATACG-3′ and downstream: 5′-AAAGGGAGCTGGACGCGG-3′; GLUT2 upstream: 5′-TTCCAGTTCGGCTATGACATCG-3′ and downstream: 5′-CTGGTGTGACTGTAAGTGGGG-3′.

### Western blotting

MIN6 cells, in the logarithmic growth phase, were seeded into 6-well plates at a cell density of 1 × 10^6^ cells per well. After an overnight adherent growth, the solvent control group, 0.5 μM DEX + solvent control group (referred to as the DEX group), 0.5 μM DEX + 2.5 mM 4-PBA group, and 2.5 mM 4-PBA group were set. All interventions were at 4 h, 12 h, and 24 h. The cells were washed twice with pre-chilled PBS and then added to cold RIPA lysis buffer containing 1% protease inhibitor for 10 min. The collected cell lysate was centrifuged at 4 °C to extract the total protein. From this, 30 μg of protein, mixed with the loading buffer (4:1), and heated to denaturation, were separated using a 10% acrylamide sodium dodecyl sulfate–polyacrylamide gel electrophoresis (SDS‐PAGE). Next, the protein was transferred onto polyvinylidene difluoride (PVDF) membranes which were blocked in 5% skim milk for 1 h at room temperature. Then, the primary antibodies BIP, ATF6, IRE1, PERK, CHOP Rabbit mAb (all 1:1000, CST) were incubated with the PVDF membranes at 4 °C overnight, respectively. After this incubation, the membrane was washed with TBS-T (Tris-buffered saline with Tween-20) and incubation with secondary antibody (1:5000, Boster) was carried out for 1 h. The membrane was washed again with the TBS-T for another 30 min. Finally, the protein band was visualized using the chemiluminescent reaction.

### Statistics

Data are presented as mean ± SD. Comparisons were performed using Student’s *t-*test or one-way ANOVA with Dunnett post-hoc test using the GraphPad Prism 6 program. A *P*-value < 0.05 was deemed statistically significant.

## Results

### DEX upregulates ERS-related genes in MIN6 cells

**Fig. 1:**
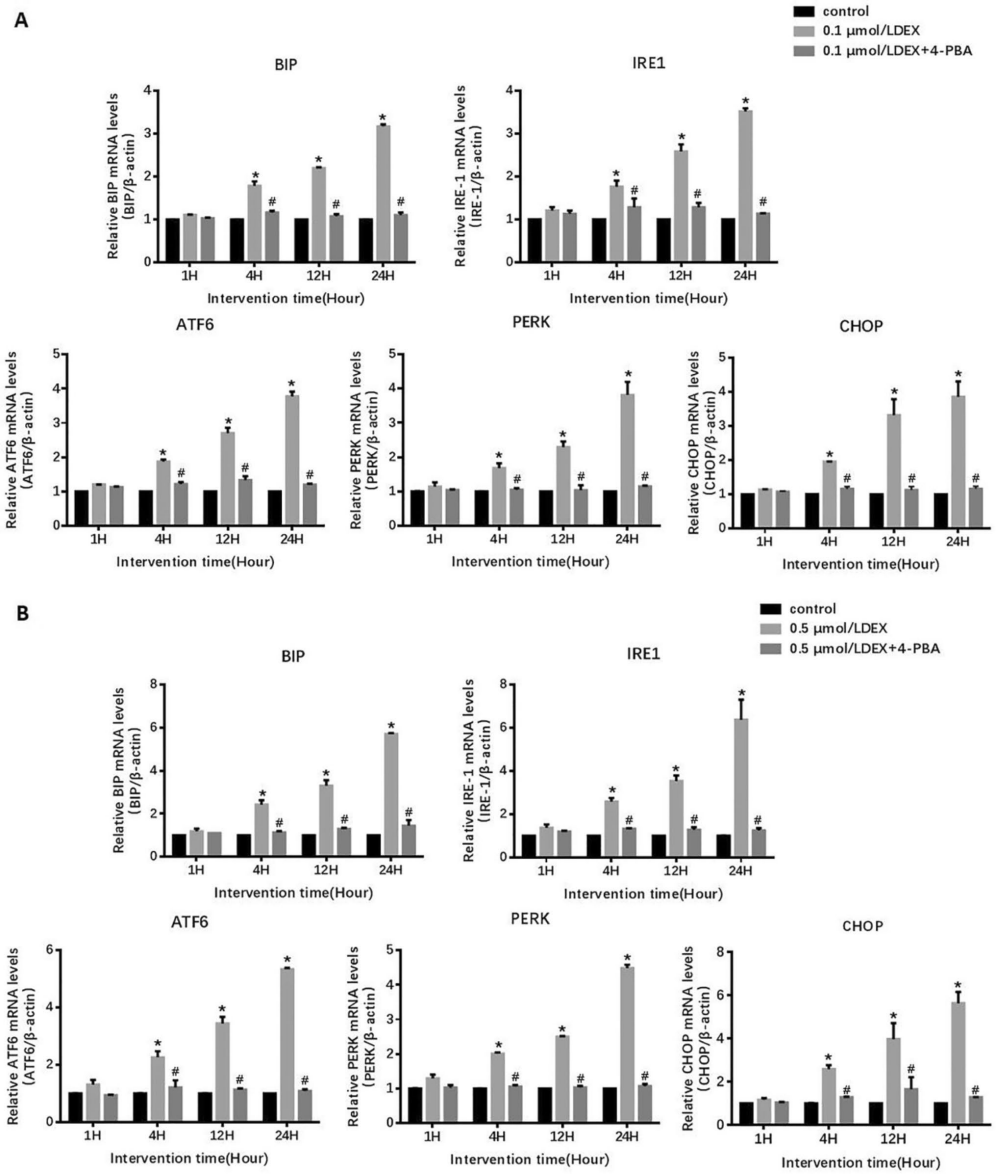
The mRNA levels of ERS-related genes in MIN6 cells after treatment with different concentration of DEX or DEX + 4-PBA. Data are expressed as a mean value ± SD of three independent experiments. **a** Treated with 0.1 μmol/L DEX in the presence or absence of 2.5 mmol/L 4-PBA. Vs the control group *(P < 0.01); Vs 0.1 μmol/L DEX group ^#^(P < 0.01). **b** Treated with 0.5 μmol/L DEX in the presence or absence of 2.5 mmol/L 4-PBA. Vs the control group *(P < 0.01); Vs 0.5 μmol/L DEX group ^#^(P < 0.01)

The qPCR results show that compared to the control group, both low and high concentrations (0.1 μmol/L and 0.5 μmol/L, respectively) of DEX intervention up-regulated the mRNA levels of unfolded protein response-related genes and *CHOP* in MIN6 cells (Fig. 1). The increased levels of *CHOP* can be directly correlated with increased cell apoptosis. As per time points analysis, DEX intervention at 4, 12, and 24-h groups were significantly higher (P < 0.01) compared to the control group (Fig. 1). Among the concentrations, mRNA levels were higher for 0.5 μmol/L DEX intervention than 0.1 μmol/L DEX intervention for 1 h, 4 h, 12 h, and 24 h in MIN6 cells. There was no statistical difference in control groups at different time. (Supplementary Fig. 1).

### 4-PBA attenuated DEX-induced ERS and apoptosis in MIN6 cells

**Fig. 2:**
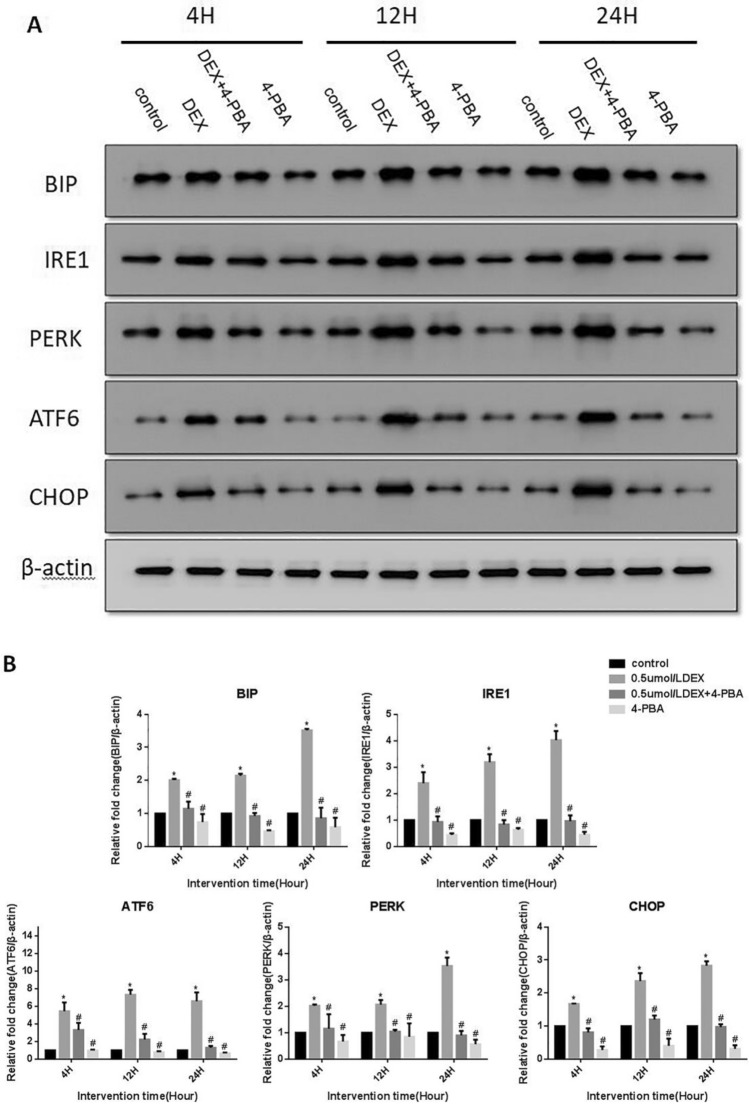
Effect of 0.5 μmol/L DEX and/or 4-PBA on the protein levels of BIP, IRE1, PERK, ATF6, and CHOP. **a** Western blotting analysis of BIP, IRE1, PERK, ATF6, and CHOP protein levels in the control and treated groups in MIN6 Cells. **b** Quantification of the relative protein levels of BIP, IRE1, PERK, ATF6, and CHOP, normalized with the internal marker β-actin (P < 0.01). Data are expressed as a mean value ± SD of three independent experiments. Vs control group *(P < 0.01); Vs 0.5 μmol/L DEX group ^#^(P < 0.01)

Since we find out that the high concentration (0.5 μmol/L) of DEX was more effective in upregulating the level of ERS-related genes (Fig. 1), we selected 0.5 μmol/L DEX for the subsequent experiments and increased the 4-PBA group. The results of western blot analysis showed that the protein levels of BIP, ATF6, IRE1, PERK, and CHOP were significantly reduced in the cells than those in the DEX group (P < 0.01). This effect was comparable to the control group without the DEX intervention and the 4-PBA group alone (Fig. 2). These results clearly indicate that DEX indeed induced ERS in MIN6 cells. However, 4-PBA treatment could reduce the DEX-induced ERS.

### Effect of DEX on GLUT2 and PDX1 genes in MIN6 cells

**Fig. 3:**
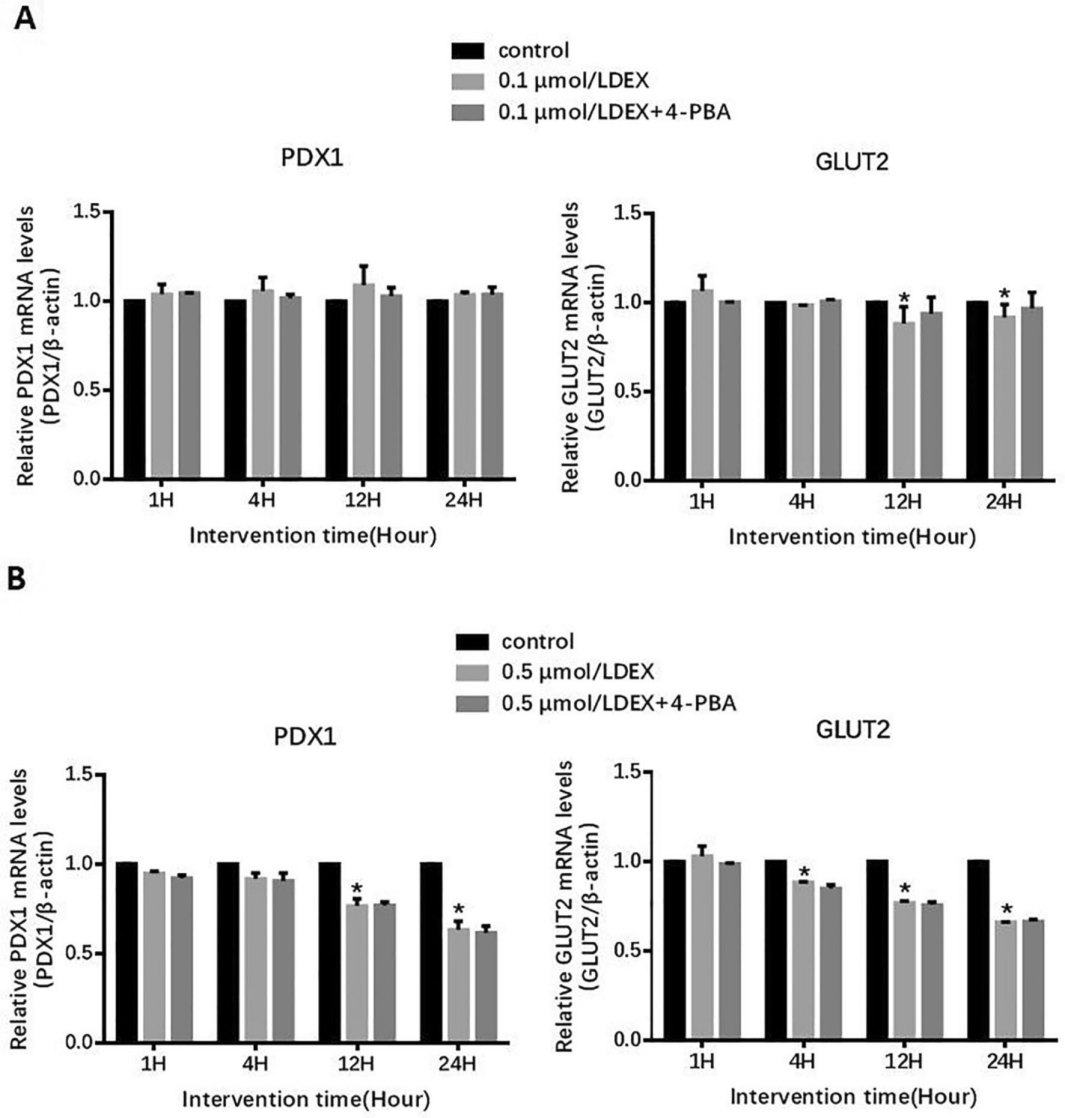
mRNA levels of *PDX1* and *GLUT2* in MIN6 Cells. **a** Treated with 0.1 μmol/L DEX and/or 4-PBA. **b** Treated with 0.5 μmol/L DEX and/or 4-PBA. Data are expressed as a mean value ± SD of three independent experiments. Vs control group *(P < 0.01)

Compared with the control group, low-concentration (0.1 μmol/L) of DEX down-regulated the mRNA level of *GLUT2* for 12 h and 24 h. Interestingly, a high-concentration (0.5 μmol/L) of DEX could reduce the *GLUT2* mRNA level even after a comparatively short 4 h intervention (P < 0.01) (Fig. 3). Similarly, low concentration (0.1 μmol/L) of DEX had little effect on *PDX1* levels in MIN6 cells. Again, here too, high concentration (0.5 μmol/L) of DEX showed marked reduction in the mRNA level of *PDX1* (Fig. 3). However, 4-PBA did not affect *PDX1* and *GLUT2* levels in DEX-induced MIN6 cells (Fig. 3).

## Discussion

In this study, firstly, we found that long-term exposure to DEX induces ERS and ERS-mediated apoptosis in MIN6 cells, both in a time and concentration-dependent manner. Interestingly, 4-PBA could alleviate these DEX-induced UPR changes in the MIN6 cells in vitro. This is evident by the simultaneous reduction of BIP and the three pathway-related genes, downstream of the ER stress response, ATF6, IRE1, and PERK to the normal control level. Moreover, 4-PBA also inhibited the DEX-induced ERS-mediated apoptosis. Concerning insulin synthesis and release, low concentrations of DEX did not affect the expression levels of *GLUT2* and *PDX1* genes in MIN6 cells. However, a high concentration of DEX markedly reduced the expression levels of these. It appears that the distinct DEX concentrations may have different degrees of effect on insulin synthesis and secretion in the β-cells. Moreover, unlike the protective effect of the up-regulation of ER related genes upon DEX treatment, 4-PBA has no significant protective effect on DEX-induced down-regulation of insulin biosynthesis-related genes. This could be since *GLUT2* and *PDX1* genes are not directly associated with ER stress, or due to the limitation of only assessing the gene expression levels rather than the secretion function of the islet cells.

GCs-induced hyperglycemia is mostly associated with progressive insulin resistance in the surrounding tissues, but recent studies also suggest the involvement of the islet β-cell dysfunction [13]. GCs mediated apoptosis leads to a reduced number of pancreatic β-cells that in turn affect insulin biosynthesis [14]. GCs also cause β-cell dysfunction by disrupting the cell redox balance leading to ER stress. In a study using INS-1E cells, it was found that prednisolone activates ER-stress ATF6 and IRE1 pathways, and simultaneously up-regulates the expression of apoptosis-related factors CHOP and caspase-3 for cell apoptosis [15]. There is no way to mimic the in vivo GC concentration precisely in vitro. Because of the different distribution of drugs in vivo, the concentration around islet cells in vivo is still unknown even though the blood concentration of GC can be measured. The effective treatment concentration in vitro study was selected based on documents and pre-tests. In this study, we found that both high and low concentrations of DEX could activate the three classical UPR pathways, including ATF6, IRE1, and PERK, and also up-regulated CHOP. However, 4-PBA showed a good inhibitory effect on DEX-induced ER stress-related proteins by downregulating CHOP both at mRNA and protein levels, which is a pro-apoptotic factor in ERS [12, 16].

If UPR activation fails to restore ER homeostasis under decompensated ERS, then UPR shifts from an adaptive program to a pro-apoptotic program. Chronic PERK activation increases the expression of CHOP, by activation of the transcription factor ATF4 (activating transcription factor 4). CHOP also plays an important role in the ER stress-induced β-cell apoptosis [17, 18]. For instance, targeted disruption in the *CHOP* gene delays the disease onset in the Akita mouse, a diabetic model related to ER stress [17]. In summary, activation of UPR reduces the synthesis and transport of newly synthesized proteins to the ER. It also simultaneously increases the folding capacity of organelles by increasing the synthesis of ER molecular chaperones along with increased processing of the irreversibly misfolded proteins.

ERS is an important factor in the pathogenesis of diabetes. Therefore, prevention or reduction of ERS in β-cells could be a novel strategy for the prevention and/or treatment of diabetes. Moreover, 4-PBA has shown to improve the inhibition of palmitate-induced glucose-stimulated insulin secretion (GSIS) in primary rat islets. It does so by reducing the ER stress in obese mice to restore normal insulin sensitivity and blood glucose [19]. Interestingly, in humans too, PBA has shown to improve insulin resistance and β-cell dysfunction that resulted from the prolonged elevation of free fatty acids. These effects were due to PBA dependent reduction in ERS [20]. As an inhibitor of ER stress, 4-PBA eliminates intracellular UPR, and inhibits ER stress by regulation of several vital proteins, including BIP, PERK, ATF6, IRE1 and CHOP. Upregulation of some ER stress markers, especially BIP and CHOP, was observed in MIN6 cells after DEX intervention, suggesting that ER stress was induced by DEX. In summary, our study demonstrated that suppression of ER stress with 4-PBA inhibits GC-induced apoptosis by attenuating ER stress. We also observed that 4-PBA alone inhibit expression of CHOP, PERK, ATF6, IRE1 and BIP at 24 h, some even at 12 h (CHOP). 4-PBA has non-specific effects only in the presence of DEX. But DEX mediated ER stress is more obvious, and 4-PBA can alleviate this effect. We hope to provide a novel clue for molecular intervention against DEX-induced islet cell damage by inhibiting ER stress. Therefore, 4-PBA can be considered as a new therapy for diabetes. In our study too, we show that PBA relieves GCs-induced β-cell damage by alleviating the ER stress. However, the effect of 4-PBA on β-cell function remains to be studied further.

## Supplementary Information

Below is the link to the electronic supplementary material.

Supplementary material 1 (DOCX 24  kb)
