# Supplementary material for: 4-Phenylbutyric acid protects islet β cell against cellular damage induced by glucocorticoids
Source: Mol Biol Rep. 2021 Feb 10;48(2):1659–65. doi: 10.1007/s11033-021-06211-5 (PMC7925466; doi:10.1007/s11033-021-06211-5)
Supplement: Supplementary file 1 — Supplementary material 1 (DOCX 24 kb) [file 11033_2021_6211_MOESM1_ESM.docx]

1、The controls, at 4, 12, and 24 hr were compared to the control condition at 1 hour since in vitro incubation itself may activate the ERS genes.There was no statistical difference as shown in the figure below.

Supplementary Figure 1.

2、Preparation for DEX and 4-PBA:

(1) Preparation of DEX solution

DEX storage solution: Dissolve 5 mg of DEX in 50 ml of absolute ethanol, gently stir to dissolve, prepare a storage solution with a concentration of 0.1 mg/ ml, and it was store at -20°C.

DEX working solution: 392µL of 0.1 mg/ml DEX stock solution was added to 608 µL sterile medium to prepare 0.1 mmol/L DEX and it was stored at 4°C. DEX working solution is diluted as needed and filtered for later use.

(2) Preparation of 4-phenylbutyric acid solution

4-PBA was dissolved in DMSO to prepare a 2 g/ml 4-phenylbutyric acid solution. Take 20.5 µL of 2 g/ml PBA solution and add 10 ml of sterile medium to make 25 mmol/L 4-PBA solution, adjust the pH to 7.0-7.4, and reserve.

Working solution 4-PBA concentration (2.5 mmol/L): 25 mmol/L 4-PBA solution was diluted 10 times to obtain 2.5 mmol/L 4-PBA solution, which was filtered for use.
